# Bibliometric analysis of targeted immunotherapy for osteosarcoma-current knowledge, hotspots and future perspectives

**DOI:** 10.3389/fimmu.2024.1485053

**Published:** 2025-02-10

**Authors:** Yunxiang Hu, Rui Yang, Shuai Ni, Zefeng Song

**Affiliations:** ^1^ Department of Orthopaedic Trauma, The Second Affiliated Hospital of Dalian Medical University, Dalian, Liaoning, China; ^2^ School of Graduates, Dalian Medical University, Dalian, Liaoning, China; ^3^ School of Graduates, Dalian University of Technology, Dalian, Liaoning, China

**Keywords:** osteosarcoma, immune function, immunotherapy, immune checkpoint inhibitor, targeted therapy

## Abstract

**Objective:**

The objective of this study is to conduct a bibliometric analysis on examining the current condition, areas of interest, and rising trends of immunotherapy for osteosarcoma (ITFOS), as well as its importance in associated research domains.

**Methods:**

An extensive collection of academic papers on the use of ITFOS was obtained from the Web of Science between January 1, 2000 and October 20, 2023. Then, using a variety of tools like HisCite, VOSviewer, CiteSpace, and the bibliometrix package, a bibliometric study was carried out. This study included the collection of information on country, institution, author, journal, and keywords.

**Results:**

A comprehensive analysis was undertaken on a total of 616 publications obtained from 247 journals, encompassing the contributions of 3725 authors affiliated with 831 institutes spanning across 43 countries/regions. Notably, China exhibited the highest quantity of published 277 (44.99%) articles on ITFOS. The most productive institution was Zhejiang University, with 26 (4.22%) publications. The author with the highest publication output was Tsukahara, Tomohide from Japan with 15 (2.44%) publications. The article with the most citation was “DOI: 10.1200/JCO.2014.58.0225”. Frontiers in Immunology demonstrated the highest level of productivity, having published a total of 31 (5.03%) articles. The most frequently used were “osteosarcoma,” “immunotherapy,” and “cancer,”. Meanwhile, “sequencing”, “prognostic signature” and “immune microenvironment“ have been identified as the research frontiers for the forthcoming years.

**Conclusion:**

This paper provides a thorough evaluation of current research trends and advancements in ITFOS. It includes relevant research findings and emphasizes collaborative efforts among authors, institutions, and countries.

## Introduction

Osteosarcoma is the most common primary malignant bone tumor characterized by highly aggressive and metastatic behavior, accounting for approximately 56% of primary malignant bone tumors (1). It predominantly affects children and adolescents with a median age of 16 years and occurs most frequently in the metaphysis of long bones, including the distal femur and proximal tibia (2). According to the 5th edition of the World Health Organization (WHO) classification of bone and soft tissue tumors, osteosarcoma is classified into several subtypes, including low-grade central osteosarcoma, conventional osteosarcoma, telangiectatic osteosarcoma, small cell osteosarcoma, parosteal osteosarcoma, periosteal osteosarcoma, high-grade surface osteosarcoma, and secondary osteosarcoma (3). Surgical intervention is an important treatment modality for osteosarcoma. With the introduction of neoadjuvant chemotherapy, the combination of chemotherapy and surgery has improved the 5-year survival rate to 70% for non-metastatic osteosarcoma patients. However, for advanced and recurrent osteosarcoma patients, despite the incorporation of various chemotherapy regimens, the treatment outcomes have remained poor based on decades of research, with a 5-year survival rate of only 20% (4, 5). Consequently, there is an urgent need to explore novel treatment approaches that can fundamentally improve the prognosis of osteosarcoma. In recent years, researchers have made significant progress in the study of tumor immune responses, leading to the development of targeted therapies focusing on T cells or their receptors in the tumor microenvironment. One such example is the use of immune checkpoint inhibitors, such as PD-1/PD-L1 monoclonal antibodies, which have shown promising therapeutic effects in various types of tumors, including melanoma, lung cancer, and breast cancer (6–9). These successes have reignited the enthusiasm for tumor immunotherapy research. In the case of osteosarcoma, researchers have increasingly conducted basic research and clinical trials exploring the potential of immunotherapy due to the challenges faced in achieving standardized treatment for osteosarcoma patients.

In the field of science, bibliometric analyses are frequently used to evaluate published research and predict future trends. The field of bibliometrics examines the relationships between scientific fields, countries, organizations, authors, and publications by using mathematical and statistical approaches (9, 10). In recent years, significant advancements have been made in the study of ITFOS, yet a bibliometric analysis of this research is lacking. This study aims to conduct a bibliometric analysis of ITFOS research. By utilizing knowledge maps, scientists can efficiently analyze large datasets and gain insights into the development and emerging trends in this field. This methodology enhances the ability to identify research hotspots and allows for a comprehensive examination of research patterns. Furthermore, the analysis may offer valuable insights for future research projects and decision-making processes.

## Materials and methods

### Search strategy

Using the Web of Science Core Collection (WoSCC), a literature search was carried out at Dalian Municipal Central Hospital on October 20th, 2023. Use the following search parameters to find results: (((TS = (Osteosarcoma OR Osteosarcomas OR Osteosarcoma Tumor OR Osteosarcoma Tumors OR Tumor, Osteosarcoma OR Tumors, Osteosarcoma OR Sarcoma, Osteogenic OR Osteogenic Sarcomas OR Sarcomas, Osteogenic OR Osteogenic Sarcoma)) AND TS=(immunotherapy)) AND DT= (Article OR Review)) AND LA=(English). Articles that mentioned ITFOS or its synonyms in their title, abstract, or keywords were found as a result of the search query. Articles and reviews published between January 1, 2000, and October 20, 2023 were the only document kinds included in the search; publications earlier than January 1, 2000, case reports, meeting abstracts, editorial materials, and other document types were not included. Documents written in the English language were the only ones that met the inclusion criterion.

### Data collection

On October 20, 2023, a literature search query was performed in order to retrieve data from the WoSCC. The information retrieved covered a wide range of features of the literature, including authorship, title, source, sponsorship, citation count, accession number, abstract, address, document type, and cited references. To aid further analysis, the data was collected in both txt and BibTex formats. Web of Science was used to obtain the H-index of the top 10 authors with the most publication output. Furthermore, the 2022 impact factor and Journal Citation Report category quartile of the ten key journals relevant to ITFOS were obtained from Web of Science.

### Statistical analysis

HisCite (version 12.03.17), VOSviewer (version 1.6.18), CiteSpace (version 6.1.R3), and the bibliometrix package (version 3.2.1; https://cran.r-project.org/web/packages/bibliometrix/) based on R language (version 4.1.2) were used to analyze the bibliometric data. HisCite was used to calculate the total number of publications and citations for producing countries, institutions, and authors. In addition, using HisCite, the top ten papers with the highest citation count in ITFOS were discovered. The yearly count of publications was calculated using HisCite and graphically represented in the R programming language using the ggplot2 package (version 3.3.6; https://github.com/tidyverse/ggplot2). VOSviewer was used to identify the top 10 keywords with the highest occurrence, bibliometric coupling within journals, and clustering of the top 54 keywords. CiteSpace was also used to create a dual-map overlay of the journals connected with ITFOS. CiteSpace was used to assess the level of collaborative centrality among countries/regions, institutions, and authors. Following that, trend topic detection within the bibliomatrix program was used as an alternate methodology. This program was also used to build visual representations of publication volume and collaborative relationship networks. When analyzing different subjects, it’s important to choose appropriate tools based on their specific characteristics. For example, in clustering diagrams for keywords, countries, and authorses,sti algorithmic influence is minimalcei focus on the compactness and aesthetics, making VOSviewer a suitable choice. In contrast, Citespace is better for citation bursts, co-citation networks, and timelines. The bibliometrix R package provides intuitive visualizations for trend topics, while the interactive tool biblioshiny has limited interactivity. However, bibliometrix also allows for custom visualizations, such as keyword time heatmaps. These tool differences necessitate careful selection based on research objectives. To mitigate discrepancies from using different tools, we analyze identified trends or clusters with multiple tools and compare the results. If consistent trends are found across tools for the same keyword or topic, we consider these findings robust. In our final reports, we prioritize keywords or topics identified as hotspots across various analyses to ensure the reliability and generalizability of the results.

## Results

### Overview

**Figure 1:**
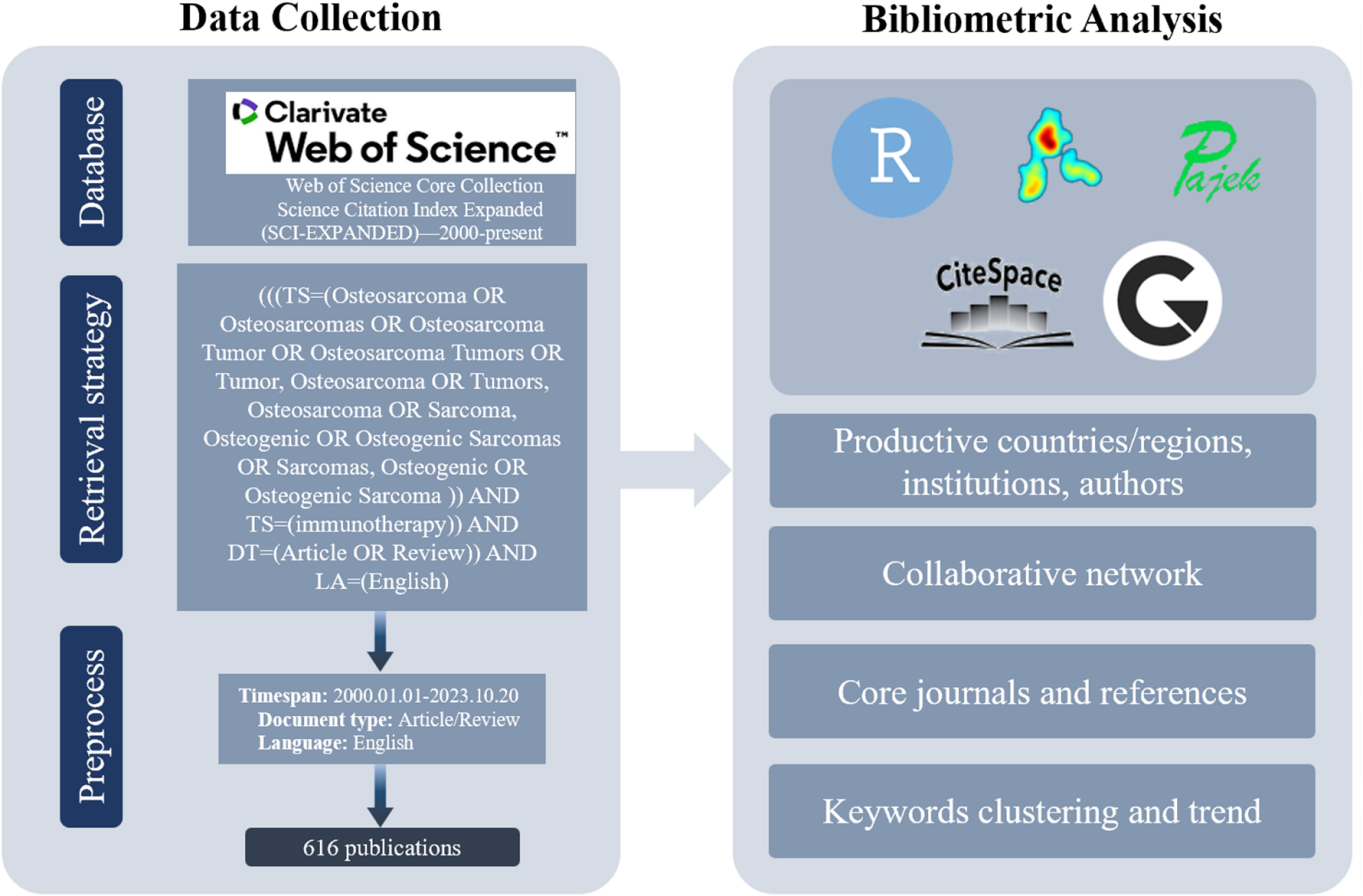
Hierarchical chart depicting the process of publication selection.

A comprehensive search was conducted in the WoSCC database, resulting in the identification of 616 publications pertaining to ITFOS. The search period spanned from January 1, 2000, to October 20, 2023 (**Figure 1**).

**Figure 2:**
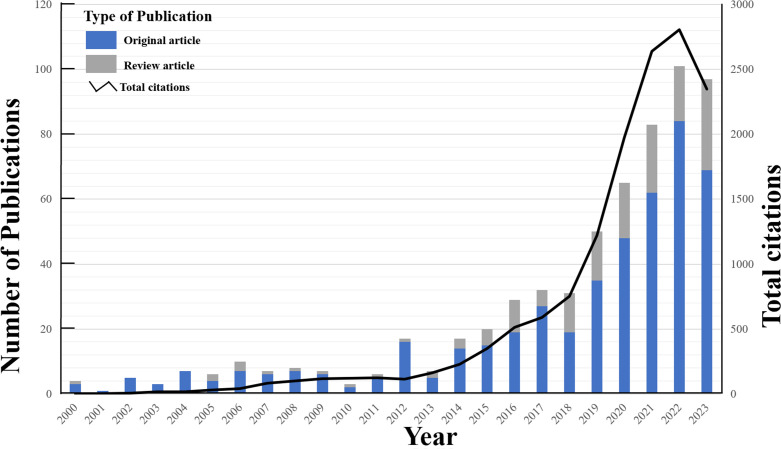
The yearly quantity and citations of publications pertaining to ITFOS.

Among these publications, 469 were categorized as original articles, while 147 were classified as review articles. Notably, the frequency of ITFOS-related publications exhibited an irregular pattern, albeit showing an overall upward trend in terms of total citations (**Figure 2**).

It is worth mentioning that the proportion of original articles consistently surpassed that of review articles on an annual basis. The cumulative collection of published articles has garnered a total of 14355 citations, resulting in an average of 23.31 citations per article, which holds significant scholarly value.

### Leading countries/regions

**Figure 3:**
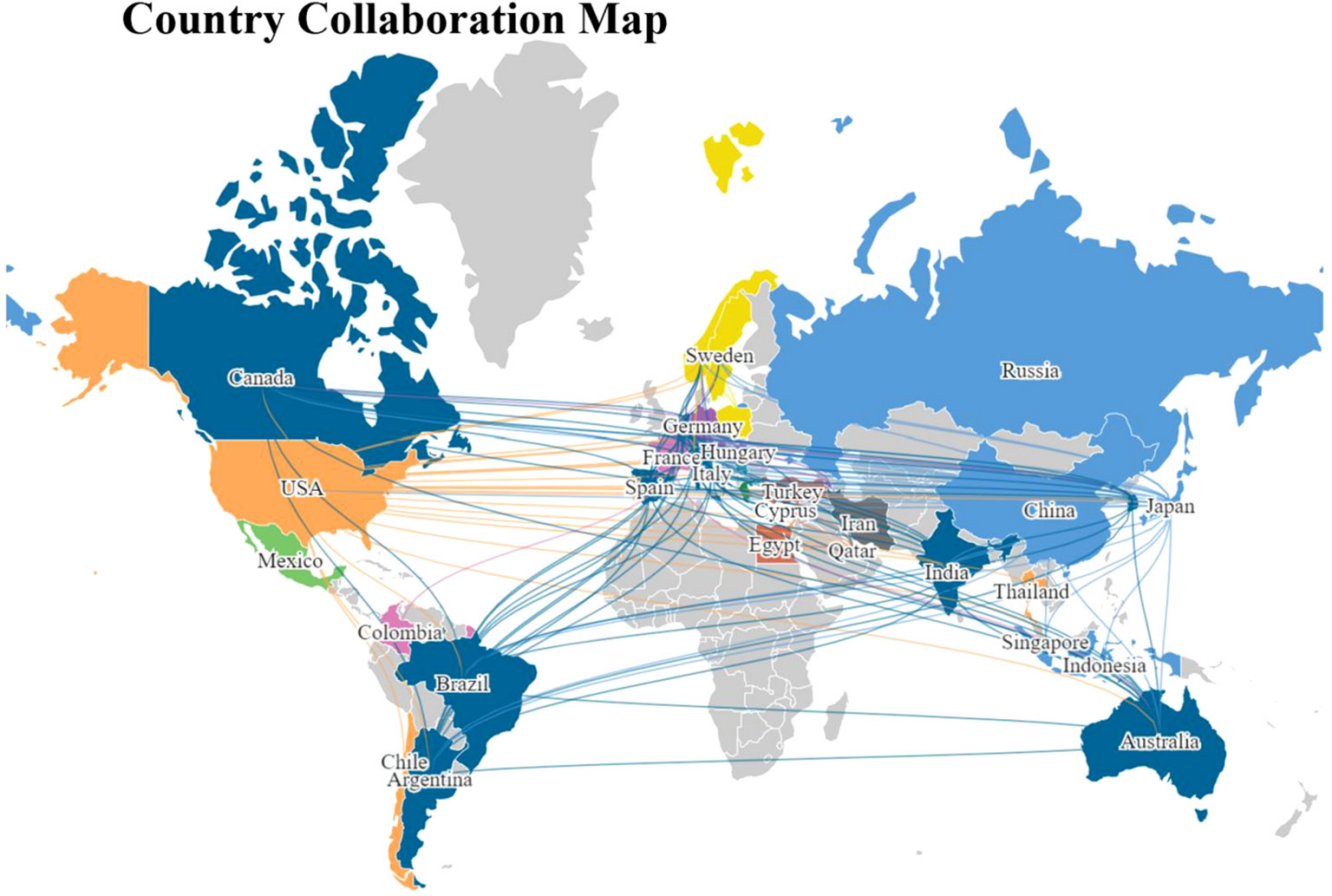
The collaborative map of the country.

From January 1, 2000, to October 20, 2023, scholarly articles on ITFOS were disseminated across 43 countries/regions spanning six continents. Notable collaboration was observed among East Asia, North America, and Western Europe (**Figure 3**).

This study highlights the top 10 countries in terms of productivity in publishing articles on ITFOS. China emerged as the most prolific country, contributing 277 (44.99%) articles, followed by USA with 192 (31.17%) articles, Japan with 68 (11.04%) articles, Italy with 32 (5.19%) articles, and Germany with 25 (4.06%) articles. It is noteworthy that articles originating from the United States received the highest total number of citations, amounting to 6826, while articles from Germany had the highest average number of citations per article, with 57.16 (**Table 1**).

**Table 1:** The top 10 countries/regions with the highest productivity.

Rank	Country	Publications n(%)	Total citations	Average citations	Collaborative centrality
1	China	277 (44.99%)	3964	14.31	0.00
2	USA	192 (31.17%)	6826	35.55	0.22
3	Japan	68 (11.04%)	1992	29.29	0.06
4	Italy	32 (5.19%)	762	23.81	0.02
5	Germany	25 (4.06%)	1429	57.16	0. 12
6	United Kingdom	24 (3.90%)	937	39.04	0.13
7	France	21 (3.41%)	722	34.38	0.02
8	Australia	13 (2.11%)	309	23.77	0. 01
9	Netherlands	12 (1.95%)	607	50.58	0.03
10	Spain	12 (1.95%)	306	25.50	0. 04

### Active institutions and authors

Through an extensive investigation, a total of 616 publications were identified, authored by 3725 individuals affiliated with 831 institutes across 43 countries/regions. Among the identified institutes, Zhejiang University in the China emerged as the most prolific, contributing 26 (4.22%) publications. This was followed by the Central South University, China with 22 (3.57%) publications, University of Texas MD Anderson Cancer Center, USA with 21 (3.41%) publications, Sapporo Medical University in Japan with 20 (3.25%) publications, and Shanghai Jiao Tong University, China with18 (2.92%) publications. Notably, among the top ten institutions, six were located in the China, three in USA, and one in Japan (**Table 2**).

**Table 2:** The top 10 productive institutions.

Rank	Institution	Country	Publications n(%)	Total citation	Average citation
1	Zhejiang University	China	26 (4.22%)	709	27.27
2	Central South University	China	22 (3.57%)	118	5.36
3	University of Texas MD Anderson Cancer Center	USA	21 (3.41%)	1480	70.48
4	Sapporo Medical University	Japan	20 (3.25%)	373	18.65
5	Shanghai Jiao Tong University	China	18 (2.92%)	330	18.33
6	Memorial Sloan Kettering Cancer Center	USA	17 (2.76%)	798	46.94
7	Peking University	China	14 (2.27%)	358	25.57
8	Fourth Military Medical University	China	13 (2.11%)	186	14.31
9	Sun Yat-sen University	China	13 (2.11%)	177	13.62
10	National Cancer Institute (NCI)	USA	12 (1.95%)	595	49.58

**Figure 4:**
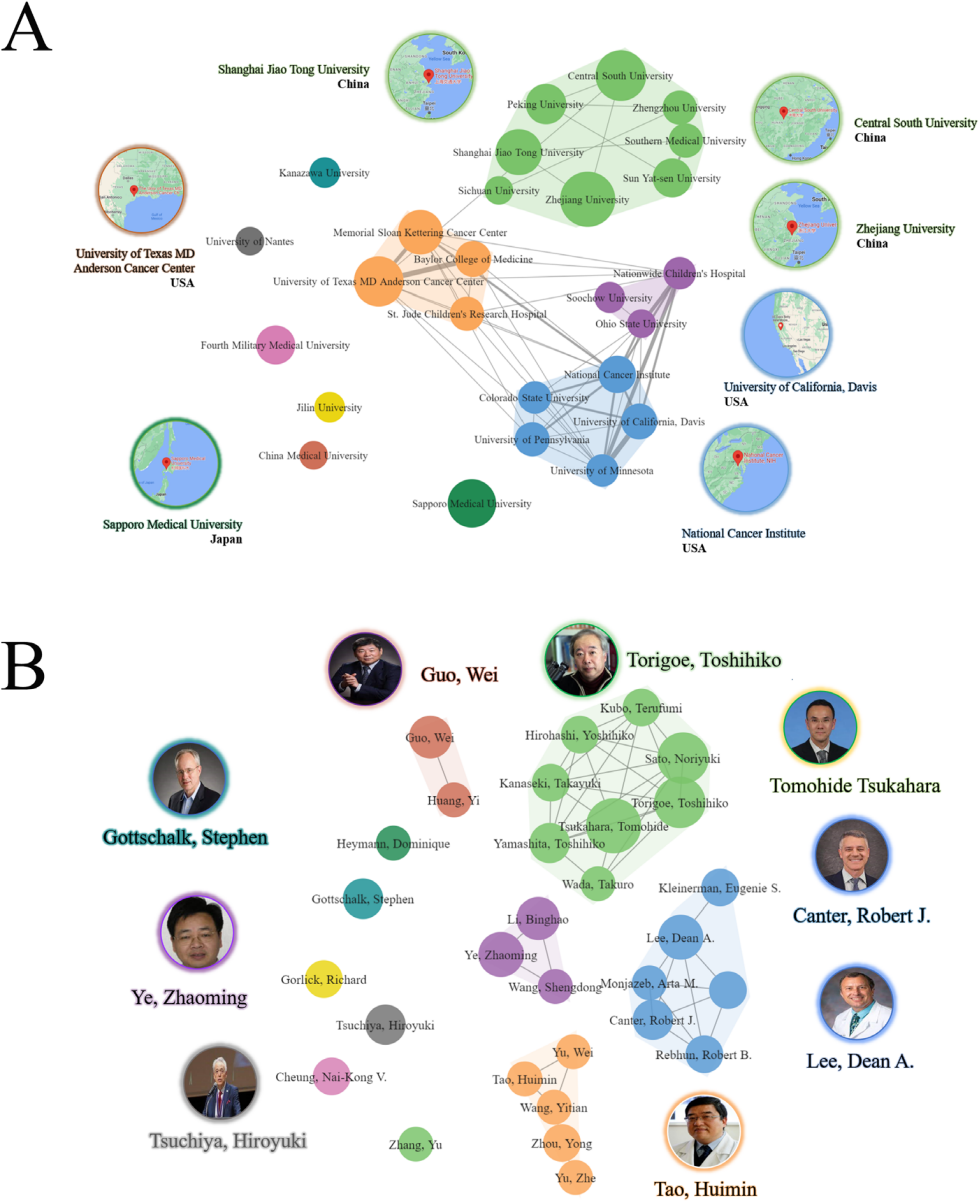
Collaborative clustering of institutions and authors. **(A)** Collaborative clustering of institutions. **(B)** Collaborative clustering of authors.

The study also revealed four distinct clusters of institutional collaboration, with Zhejiang University, University of Texas MD Anderson Cancer Center, Nationwide Children’s Hospital and National Cancer Institute (NCI) exhibiting the highest level of collaboration (**Figure 4A**). Regarding authors, the most prolific author identified was Tsukahara, Tomohide, Japan with 15 (2.44%) publications, followed by Sato, Noriyuki, Japan with 13 (2.11%) publications, and Torigoe, Toshihiko, Japan, with 13 (2.11%) publications. Among the top ten most productive authors, four were affiliated with institutions in Japan, two with institutions in China, one with an institution in USA, one with an institution in Germany, and one with an institution in Australia. Notably, Gottschalk, Stephen from Germany had the highest H-index of 64 (**Table 3**).

**Table 3:** Top 10 productive authors.

Rank	Author	Institution	Country	Publications n(%)	Total citation	Average citation	H-index
1	Tsukahara, Tomohide	Sapporo Medical University	Japan	15 (2.44%)	272	18.13	29
2	Sato, Noriyuki	Fukushima Univ Child Mental Hlth	Japan	13 (2.11%)	267	20.54	42
3	Torigoe, Toshihiko	Sapporo Medical University	Japan	13 (2.11%)	246	18.92	42
4	Lee, Dean A.	Nationwide Children’s Hospital	USA	10 (1.62%)	314	31.40	47
5	Ye, Zhaoming	Zhejiang University School of Medicine	China	10 (1.62%)	320	32.00	28
6	Guo, Wei	Peking University	China	9 (1.46%)	369	41.00	19
7	Li, Binghao	University of New South Wales Sydney	Australia	9 (1.46%)	292	32.44	22
8	Canter, Robert J.	University of California Davis	USA	8 (1.30%)	258	32.25	36
9	Gottschalk, Stephen	St. Jude Children’s Research Hospital	Germany	8 (1.30%)	325	40.63	64
10	Tsuchiya, Hiroyuki	Kanazawa University	Japan	8 (1.30%)	278	34.75	41

The authors demonstrated a notable level of cooperation, as evidenced by the presence of five clusters. Torigoe, Toshihiko, Tsukahara, Tomohide, Canter, Robert J., Tao Huimin, and Lee, Dean A. displayed a significant degree of collaborative centrality (**Figure 4B**).

### Core journals and references

A total of 43 journals have published research on ITFOS. Among these journals, Frontiers in Immunology demonstrated the highest productivity, publishing 31 (5.03%) articles related to ITFOS. This was followed by Frontiers in Oncology with 25 (4.07%) articles, Cancers with 20 (3.25%) articles, Journal for Immunotherapy of Cancer with 15 (2.44%) articles, and International Journal of Molecular Sciences with 11 (1.79%) articles. Notably, Clinical Cancer Research achieved the highest average citation rate, with an average of 74.40 citations per article (**Table 4**).

**Table 4:** Top 10 core journals.

Rank	Journal	Publications n(%)	Total citations	Average citations	2022 JCR category quartile	2022 IF
1	Frontiers in Immunology	31 (5.03%)	340	10.97	Q1	7.3
2	Frontiers in Oncology	25 (4.07%)	432	17.28	Q2	4.7
3	Cancers	20 (3.25%)	180	9.00	Q1	5.2
4	Journal for Immunotherapy of Cancer	15 (2.44%)	573	38.20	Q1	10.9
5	International Journal of Molecular Sciences	11 (1.79%)	257	23.36	Q1	5.6
6	Clinical Cancer Research	10 (1.62%)	744	74.40	Q1	11.5
7	Journal of Bone Oncology	10 (1.62%)	172	17.20	Q2	3.4
8	Oncoimmunology	10 (1.62%)	231	23.10	Q1	7.2
9	Cancer Immunology Immunotherapy	9 (1.46%)	339	37.67	Q1	5.8
10	Oncology Letters	9 (1.46%)	106	11.78	Q3	2.9

**Figure 5:**
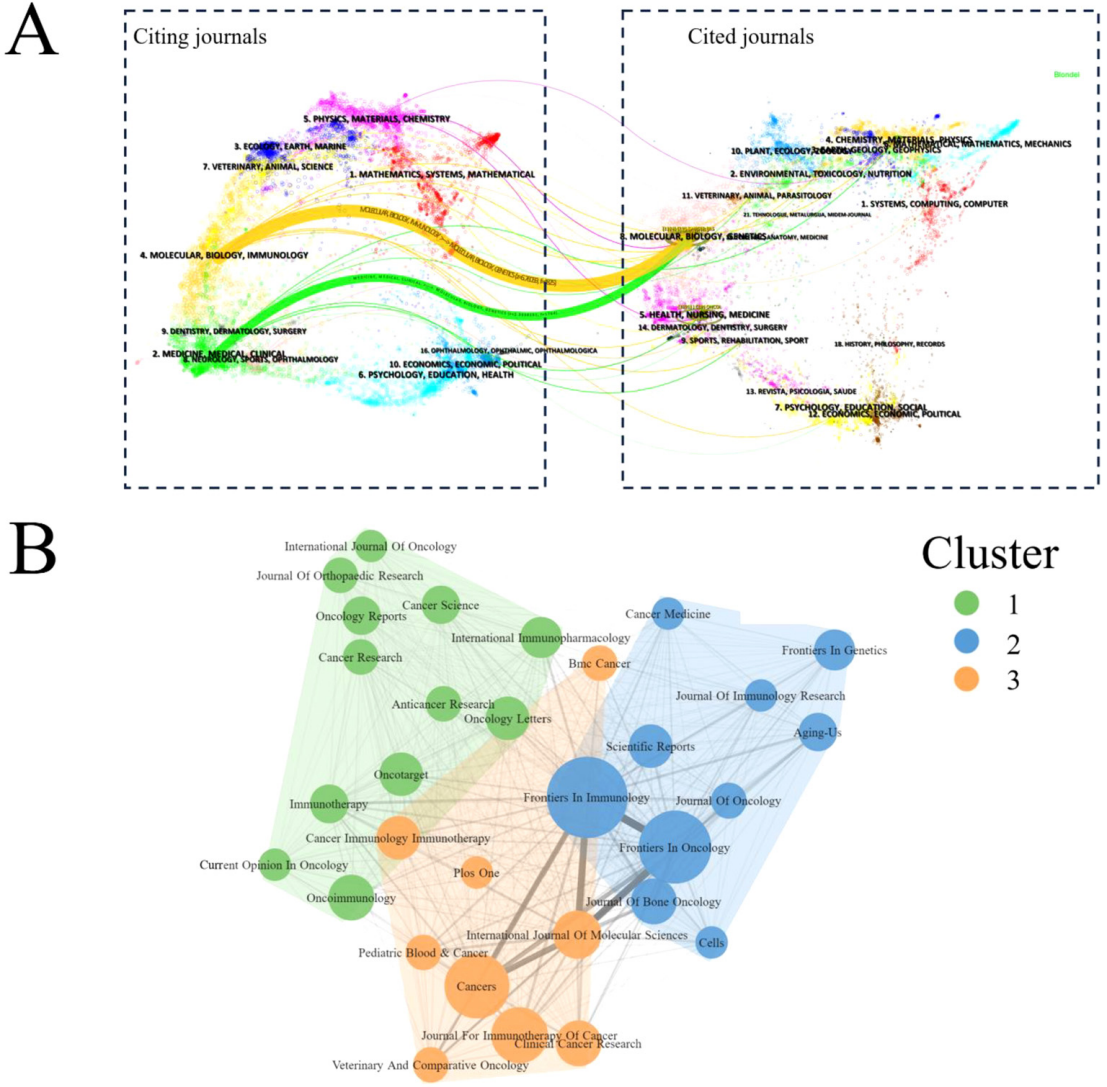
Analysis of journals. **(A)** The dual-map overlay of journals publishing studies on ITFOS. Citing journals are on the left, cited journals are on the right, and lines represent the citation relationship. **(B)** Bibliometric coupling within journals. Three clusters were identified based on journals that had published more than five articles.

The dual-map overlay revealed a single citation pathway among the numerous inter-domain linkages between journals. Interestingly, publications in Molecular/Biology/Immunology were primarily referenced by publications in Molecular/Biology/Genetics (**Figure 5A**). Bradford’s Law, a bibliometric principle, describes the distribution of scientific literature in a specific field. It suggests that a few core information sources or journals contribute significantly to the published research in that field. In the context of ITFOS research, three clusters were identified, Frontiers in Immunology, Cancers, and Oncoimmunology emerged as the top three influential journals (**Figure 5B**).

**Figure 6:**
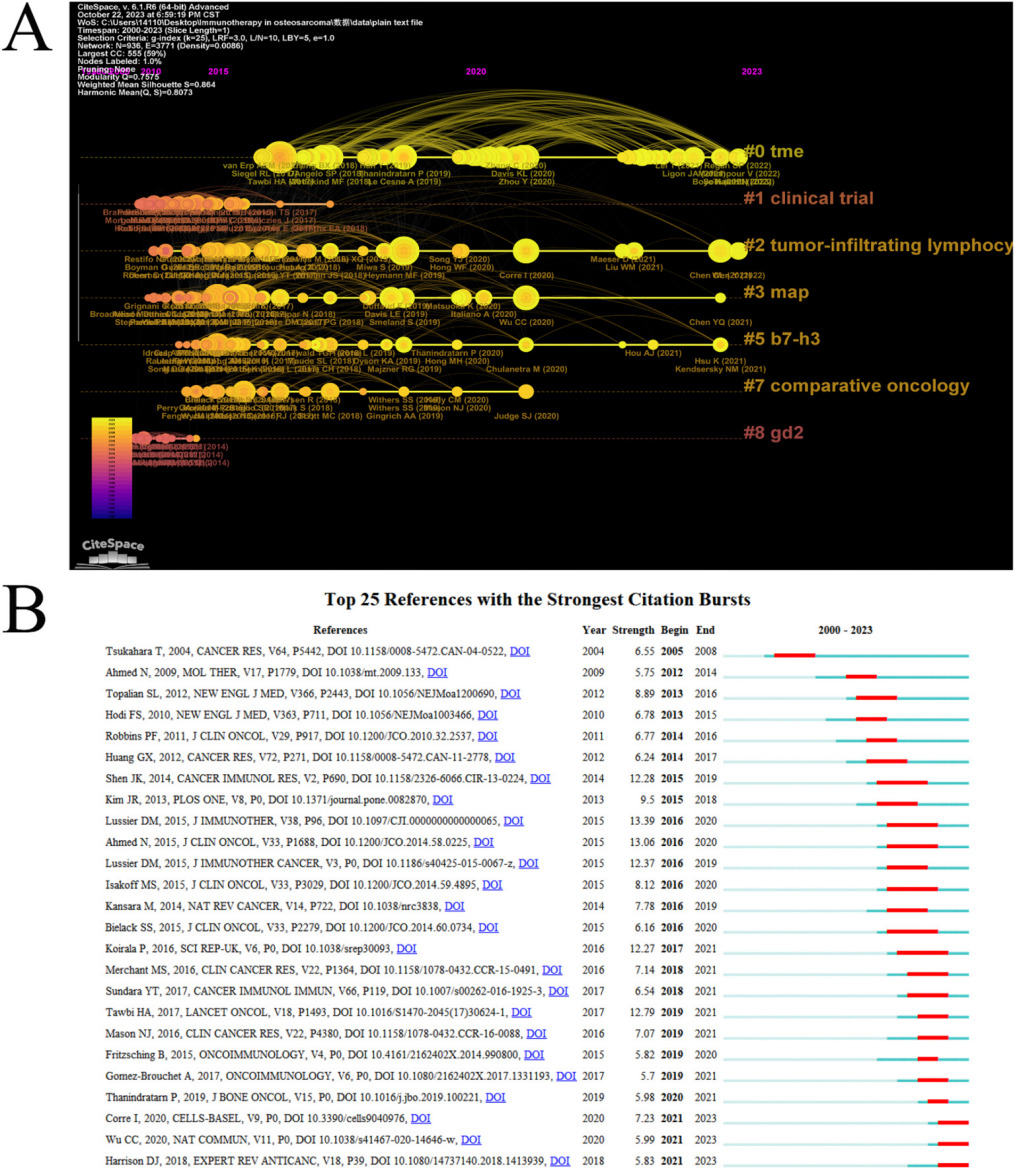
Analysis of citations and references. **(A)** Timeline of co-cited references related to ITFOS. **(B)** Reference burst detection of the top 25 references with the strongest emergent strength.

Clusters located on the right-hand side, characterized by a higher incidence of red nodes, indicate a greater prevalence of recent references. The clusters labeled “#0” (TME) and “#2” (tumor-infiltrating lymphocytes) were found to be the most temporally proximate (**Figure 6A**). A list of the top 10 papers with the most citations can be found in (**Table 5**). The article with the most citation was “DOI: 10.1200/JCO.2014.58.0225”. Ahmed et al. conducted a study that evaluated the safety and efficacy of HER2-CAR T cells in cancer patients. They found that these cells can persist for 6 weeks without significant toxicities, paving the way for future studies that combine HER2-CAR T cells with other immunomodulatory approaches to enhance their expansion and persistence (11).

**Table 5:** Top 10 core literatures.

Rank	First author	Title	Journal	Type	Year of publication	Total citations
1	Ahmed et al (11)	Human Epidermal Growth Factor Receptor 2 (HER2) -Specific Chimeric Antigen Receptor-Modified T Cells for the Immunotherapy of HER2-Positive Sarcoma	Journal of Clinical Oncology	Article	2015	719
2	Majzner et al (12)	CAR T Cells Targeting B7-H3, a Pan-Cancer Antigen, Demonstrate Potent Preclinical Activity Against Pediatric Solid Tumors and Brain Tumors	Clinical Cancer Research	Article	2019	301
3	Nakatsuka et al (13)	Immunohistochemical detection of WT1 protein in a variety of cancer cells	Modern Pathology	Article	2006	258
4	Bishop et al (14)	Future directions in the treatment of osteosarcoma	Current Opinion in Pediatrics	Review	2016	229
5	Koirala et al (15)	Immune infiltration and PD-L1 expression in the tumor microenvironment are prognostic in osteosarcoma	Scientific Reports	Article	2016	202
6	Zhou et al (16)	Single-cell RNA landscape of intratumoral heterogeneity and immunosuppressive microenvironment in advanced osteosarcoma	Nature Communications	Article	2020	187
7	Kager et al (17)	Novel insights and therapeutic interventions for pediatric osteosarcoma	Future Oncology	Review	2017	176
8	Lu et al (18)	Treatment of Patients with Metastatic Cancer Using a Major Histocompatibility Complex Class II-Restricted T-Cell Receptor Targeting the Cancer Germline Antigen MAGE-A3	Journal of Clinical Oncology	Article	2017	172
9	Chen et al (19)	Immunotherapy for osteosarcoma: Fundamental mechanism, rationale, and recent breakthroughs	Cancer Letters	Review	2021	161
10	Travis et al (20)	IASLC Multidisciplinary Recommendations for Pathologic Assessment of Lung Cancer Resection Specimens After Neoadjuvant Therapy	Journal of Thoracic Oncology	Article	2020	174

Moreover, reference burst detection was employed to identify research frontiers and emerging reference. The study examined the top 25 references with the most robust emergent properties. The reference “ doi: 10.3390/cells9040976” (21) “ doi: 10.1038/s41467-020-14646-w” (22) and “ doi: 10.1080/14737140.2018.1413939” (23) were identified as the most emergent reference in 2023 (**Figure 6B**).

### An analysis of keywords

We presented the fifteen most frequently appearing keywords in ITFOS research after consolidating synonymous terms, with “ osteosarcoma “ being the most commonly referenced (**Table 6**).

**Table 6:** Top 15 keywords by frequency.

Rank	Keyword	Occurrence	Cluster	Centrality
1	osteosarcoma	378	2	0.05
2	immunotherapy	364	1	0.12
3	cancer	158	3	0.16
4	expression	137	2	0.11
5	survival	81	2	0.03
6	t-cell	78	1	0.10
7	chemotherapy	74	5	0.10
8	cell	73	2	0.06
9	sarcoma	63	1	0.01
10	therapy	62	3	0.05
11	prognosis	54	2	0.01
12	tumor microenvironment	53	2	0.02
13	high-grade osteosarcoma	52	1	0.08
14	open-label	52	1	0.02
15	tumor	52	3	0.02

**Figure 7:**
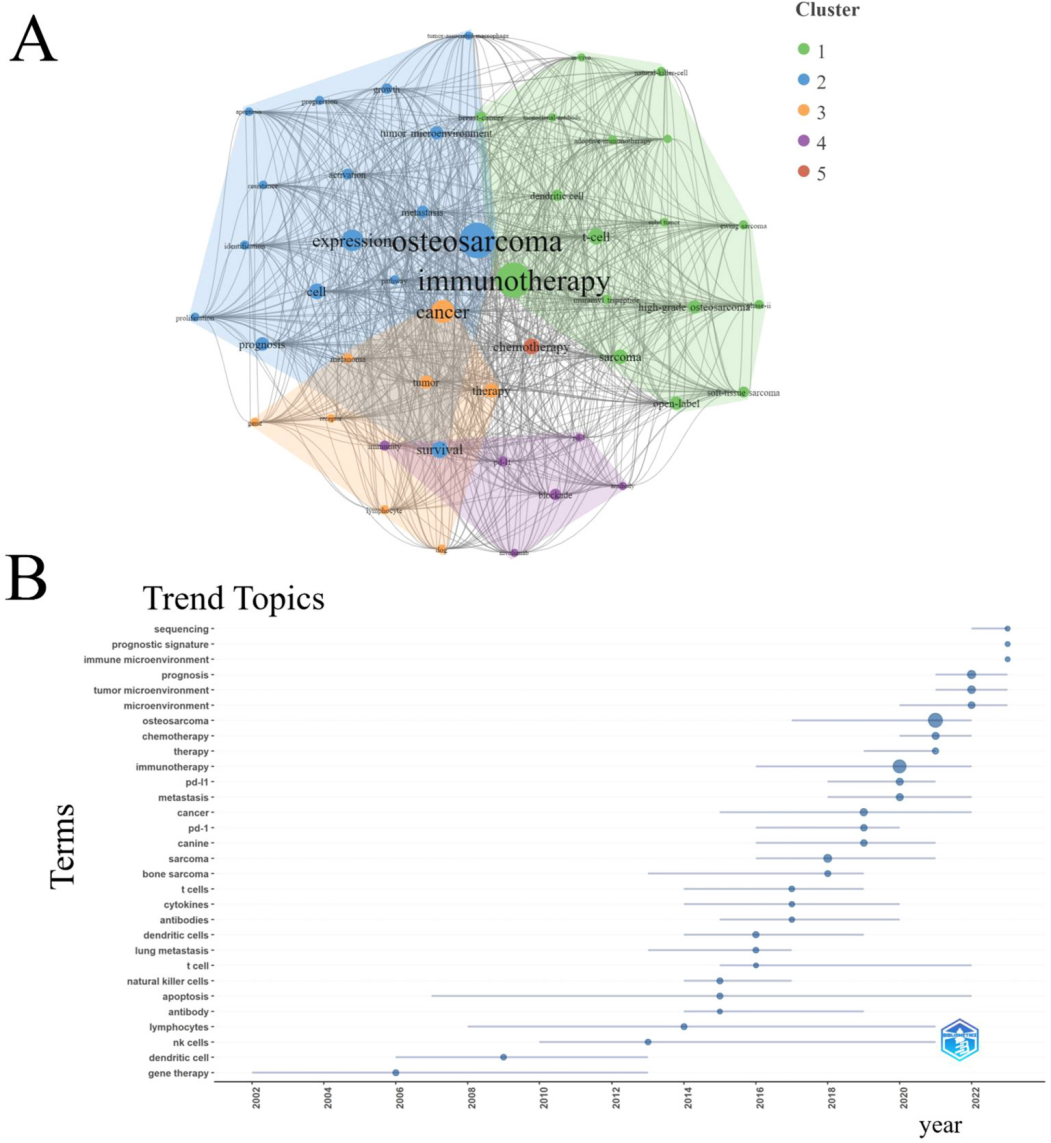
Analysis of keywords. **(A)** Clustering of the top 43 keywords with the highest number of occurrences. **(B)** Trend topics from 2000 to 2023.

An analysis of keyword co-occurrence among the top 43 keywords revealed the presence of five distinct clusters. The cluster consisting of “osteosarcoma,” “immunotherapy,” “cancer,” “chemotherapy,” and “blockade” exhibited the highest frequency of occurrence (**Figure 7A**). Furthermore, an examination of the trend topics from 2000 to 2023 identified “sequencing”, “prognostic signature” and “immune microenvironment “ as the research frontiers for the upcoming years (**Figure 7B**).

## Discussion

As early as 1891, the potential of immunotherapy for the treatment of osteosarcoma was recognized (24). In recent years, new immunotherapy approaches have emerged rapidly, and the scope of treatment has been expanding, bringing a hopeful outlook for breakthroughs in osteosarcoma treatment, which has been stagnant for several decades (25, 26). Currently, a substantial body of preclinical experiments supports the significant potential of immunotherapy in the treatment of osteosarcoma. Furthermore, several clinical trials have been initiated, involving various aspects such as T cells and immune checkpoint inhibitors (27, 28). Therefore, we conducted a bibliometric analysis and found that China exhibited the highest quantity of published 277 (44.99%) articles on ITFOS. The most productive institution was Zhejiang University, with 26 (4.22%) publications. China and Zhejiang University lead in publication counts. Possible reasons include: Osteosarcoma is a highly heterogeneous malignant tumor with a complex pathogenesis involving multiple biological processes, prompting researchers to explore it in depth. The study of osteosarcoma involves multiple disciplines, including oncology, molecular biology, immunology, and pharmacology, which fosters interdisciplinary collaboration and innovation. Advances in technology, such as genomics, transcriptomics, and immunotherapy, provide new research tools and platforms that enhance the progress of osteosarcoma research. Furthermore, international collaboration is also significant, with many research institutions and teams engaging in joint efforts in the field, sharing resources and data to facilitate rapid scientific outcomes. Additionally, increased funding support from governments and academic institutions has led to growing attention on osteosarcoma research, enabling the implementation of related projects and boosting research output. Lastly, active academic exchanges through frequent conferences and forums offer researchers opportunities to share and showcase their findings, further promoting scientific development. The study also revealed four distinct clusters of institutional collaboration, with Zhejiang University, University of Texas MD Anderson Cancer Center, Nationwide Children’s Hospital and National Cancer Institute (NCI) exhibiting the highest level of collaboration. The benefits of closer collaboration between research institutions include: Resource sharing: Collaborative institutions can share equipment, data, and funding, which enhances research efficiency and reduces costs. Improved research quality: Through collaboration, research teams can gain broader perspectives and specialized skills, thereby increasing the rigor and credibility of their research. Accelerated translation of results: Close collaboration can shorten the time required to translate research findings into practical applications, facilitating rapid technology dissemination. Enhanced impact: Collaboration enables research institutions to better expand the influence of their findings and enhance their reputation within the academic community and society at large. The author with the highest publication output was Tsukahara, Tomohide from Japan with 15 (2.44%) publications. Frontiers in Immunology demonstrated the highest level of productivity, having published a total of 31 (5.03%) articles. The most frequently used keywords were “osteosarcoma,” “immunotherapy,” and “cancer,”. The trend in keyword frequency is identified by observing its growth or decline over time, thereby marking keywords with increasing frequency (emerging trends) or decreasing frequency (declining trends). “sequencing”, “prognostic signature” and “immune microenvironment” have been identified as the research frontiers for the forthcoming years. As predicted, research on “sequencing” (29, 30), “prognostic signature” and “immune microenvironment” (16, 31–36) in osteosarcoma has gained momentum, with the number of published studies increasing annually. These topics are expected to remain prominent in the near future. Zhong et al. developed a signature (ZNF583, CGNL1, CXCL13) to predict overall survival in osteosarcoma patients, focusing on the anoikis subcluster. This signature showed strong performance in external validation, with stratification revealing significant prognostic differences. It was identified as an independent prognostic factor (29). Wu et al. found that the TMEindex is a promising biomarker for predicting prognosis in osteosarcoma patients, assessing their response to immune checkpoint inhibitor (ICI) therapy, and distinguishing molecular and immune characteristics (34). The application of immunotherapy in osteosarcoma primarily involves two aspects: enhancing the patient’s own immune system response to the tumor and exogenously boosting the immune function of the patient.

### Enhancing the patient’s immune function

The human immune system is highly complex, with various immune cells and factors working together to defend against external threats such as infections and tumors. Tumor cells can escape immune surveillance through mechanisms such as antigen concealment, downregulation of human leukocyte antigen (HLA) expression, release of inhibitory cytokines, recruitment of Treg cells, generation of bone marrow-derived suppressor cells, and promotion of tumor-associated M2 macrophages (37, 38). Therefore, enhancing the patient’s own immune function aims to eliminate tumor immune escape and reawaken the recognition of tumor cells by the body’s immune system, ultimately leading to tumor cell clearance.

### Tumor vaccines

Tumor vaccines aim to induce an anti-tumor immune response in the human body by exposing tumor antigens. Currently, the most mature technology for tumor vaccines is the cervical cancer vaccine. Cervical cancer is predominantly induced by human papillomavirus (HPV), so cervical cancer vaccines are divided into preventive vaccines (targeting HPV infection) and therapeutic vaccines (exposing tumor antigens) (39, 40). Dendritic cell (DC) vaccines, as the main type of tumor vaccine, have been applied in various types of tumor treatment to eliminate tumor cells in refractory tumors. DC vaccines are commonly used in osteosarcoma treatment (41, 42).

### Enhancing innate immunity

Recently, the role of innate immune cells in controlling tumor progression has been established. Innate immunity inhibits tumor progression by directly recognizing and killing tumor cells and triggering a strong adaptive immune response (43, 44). The cyclic GMP-AMP synthase-stimulator of interferon genes (cGAS-STING) pathway has gained significant attention due to its ability to activate the production of type I interferon, thereby enhancing anti-tumor immune responses (45). The application of STING (DMXAA, CDN, MSA-2, etc.) agonists in the treatment of osteosarcoma may be an effective strategy (46).

### Improving the tumor microenvironment

The occurrence and development of tumors are closely related to changes in the surrounding tumor tissue environment, which occurs simultaneously. Tumor cells functionally shape the tumor microenvironment by secreting various cytokines, chemokines, and other factors. This microenvironment, in turn, influences the occurrence and development of tumors, known as the tumor microenvironment. The tumor microenvironment consists of various cells (such as macrophages, neutrophils, dendritic cells, bone marrow-derived suppressor cells, NK cells, T cells, B cells, tumor-associated fibroblasts) and abundant extracellular matrix (such as collagen, fibronectin, laminin, proteoglycans), as well as various cytokines (such as IL-1β, IL-6, IFN-γ, TGF-β) (47–49). Research on the tumor microenvironment of osteosarcoma has been ongoing, and in recent years, numerous studies have demonstrated that osteosarcoma-derived extracellular vesicle (EV) play a significant role in promoting widespread immune suppression. These EV can inhibit the activity of T cells and NK cells through various pathways, and can even induce T cell apoptosis. Furthermore, they enhance the activity of bone marrow-derived suppressor cells (MDSCs) to support immune evasion by osteosarcoma cells. These research findings highlight the importance of immune suppression mechanisms in the osteosarcoma microenvironment and provide new insights for the development of therapeutic strategies targeting this microenvironment (50).

### Immune check point inhibitors

In recent years, researchers have developed targeted antibodies against cytotoxic T-lymphocyte-associated antigen 4 (CTLA-4) and programmed cell death protein 1 (PD-1) or its ligand PD-L1. These immune checkpoint inhibitors have shown promising therapeutic effects in malignant tumors such as melanoma, lung cancer (51–53). PD-1/PD-L1 as immune therapy targets have ushered in a new era of immunotherapy and accelerated research on immune treatment for osteosarcoma. Studies have shown that blocking the interaction between PD-1 and PD-L1 with antibodies significantly improves the responsiveness of osteosarcoma to cytotoxic T lymphocytes (CTLs), leading to reduced tumor burden and increased survival rates in mouse models of metastatic osteosarcoma (54). Koirala et al (15) reported a significant association between PD-L1 expression and the presence of T cells, dendritic cells, and natural killer cells in osteosarcoma. While all examined immune cell types were present in osteosarcoma samples, only infiltration of dendritic cells (28.3% vs. 83.9%, p = 0.001) and macrophages (45.5% vs. 84.4%, p = 0.031) was correlated with worse five-year event-free survival (EFS). Furthermore, PD-L1 expression was significantly associated with poorer five-year EFS (25.0% vs. 69.4%, p = 0.014). In addition, blockade of the PD-1/PD-L1 axis has also been shown to enhance the chemotherapeutic efficacy of cisplatin in osteosarcoma (55). However, there are differing opinions as well. Le et al. conducted a phase II clinical trial in 17 patients with advanced osteosarcoma and found a progression-free survival rate of only 13.3% at 6 months. They concluded that the efficacy of PD-1 inhibition in immunotherapy for osteosarcoma is limited (56). CTLA-4 is expressed on the surface of regulatory T cells (Tregs) and memory T cells. Overexpression of CTLA-4 can competitively inhibit the CD28 co-stimulatory signal required for optimal T cell activation, leading to a loss of anti-cancer activity. Additionally, binding of CTLA-4 to CD80/86 on dendritic cells can result in functional suppression of dendritic cells (57).Ipilimumab, a monoclonal antibody targeting CTLA-4 developed in 2011, was approved by the U.S. Food and Drug Administration (FDA) as the first-line immunotherapy for the treatment of melanoma (58). A phase I clinical trial demonstrated that 25% of osteosarcoma patients achieved disease stabilization following treatment with ipilimumab (59).

### Exogenous immune effector cells targeting osteosarcoma tissue

Enhancement of patient immune function through the infusion of immune cells, also known as adoptive cell-transfer therapy (ACT), is a primary approach for augmenting immune function (60). Tumor cells may downregulate the expression of their own HLA and tumor antigens, rendering them unrecognizable by T cells. Engineered T cells with high affinity for tumor-specific antigens, known as chimeric antigen receptor T cells (CAR-T cells) can recognize tumor cells independent of HLA presentation (61, 62). CAR-T cells have undergone extensive clinical trials for the treatment of hematological malignancies, achieving significant breakthroughs. In a clinical trial, CD22 CAR-T cells demonstrated an 80% response rate (24/30 patients) in the treatment of refractory or relapsed B-cell acute lymphoblastic leukemia, providing a valuable window of time for subsequent hematopoietic stem cell transplantation (63). Majzner et al (12) discovered that B7-H3 CAR T cells exhibited significant antitumor activity *in vivo*, leading to the regression of established solid tumors in xenograft models, including osteosarcoma, medulloblastoma, and Ewing sarcoma. Their findings revealed that the effectiveness of B7-H3 CAR T cells relied heavily on the high density of the target antigen on tumor tissues. Conversely, the activity of these CAR T cells was substantially reduced against target cells expressing low levels of the antigen. This observation suggests the potential for a therapeutic window, despite the low-level expression of B7-H3 in normal tissues. In osteosarcoma, primary bone tumors typically exhibit low mutation burden and are accompanied by rare naturally occurring anti-tumor T cells. Therefore, CAR-T cell therapy may be an effective strategy (64). Other adoptive cell transfer therapy approaches include CAR-NK cells and CAR-tumor infiltrating lymphocytes (TILs). Unlike T cells, NK cells are innate immune cells with cytotoxic and immunoregulatory functions (65, 66).

**Figure 8:**
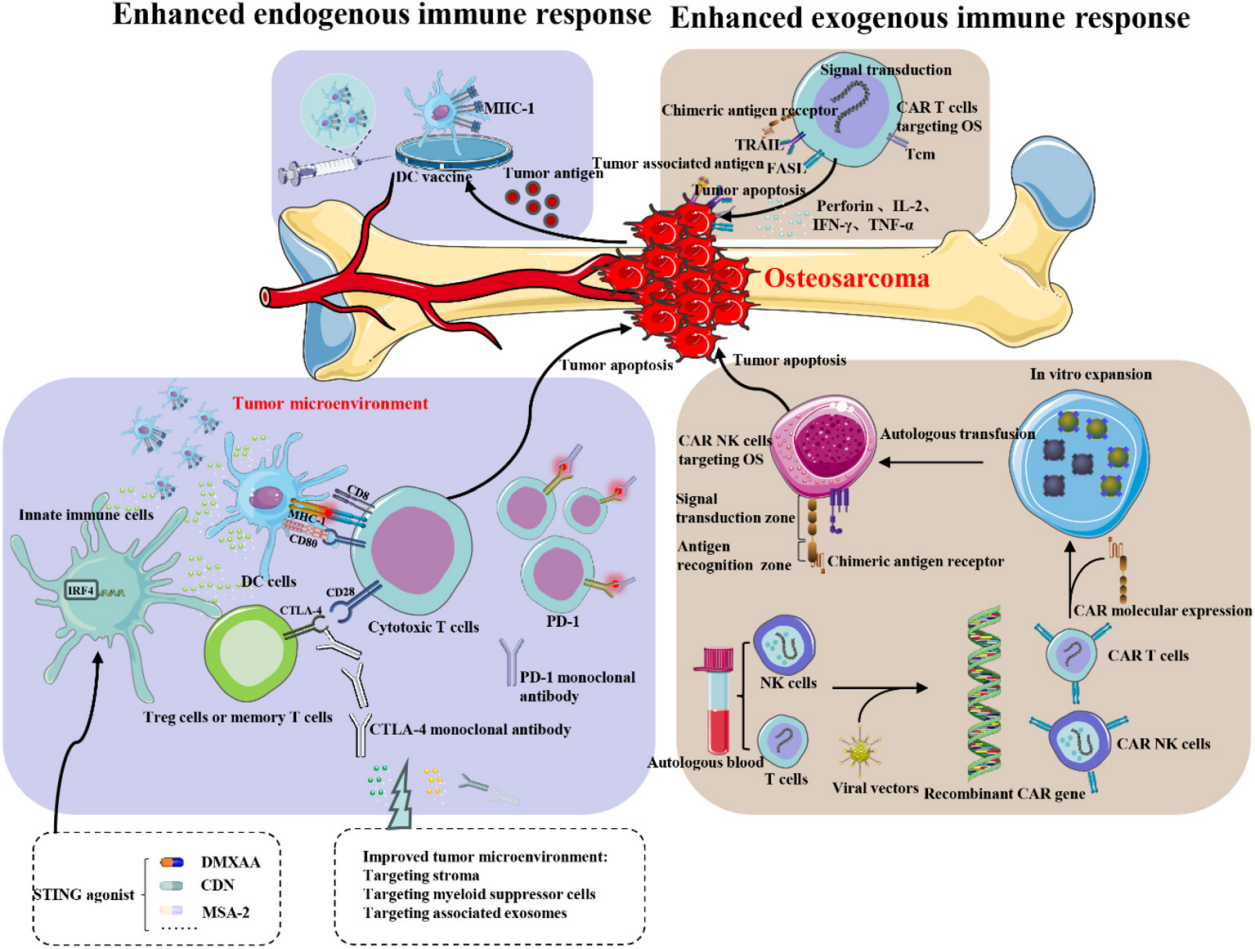
Targeted immunotherapy for osteosarcoma. The application of immunotherapy in osteosarcoma primarily involves two aspects: enhancing the patient’s own immune system response to the tumor and exogenously boosting the immune function of the patient. OS, osteosarcoma; MHC-I, major histocompatibility complex; DC cells, dendrite cells; Tcm, central memory T cell.

Since the 1970s, although there has been some improvement in the overall treatment of osteosarcoma, the therapeutic options remain limited, particularly for recurrent and metastatic osteosarcoma. Osteosarcoma cannot be cured with a single treatment. Experts from the SARC028 clinical trial indicate that resistance to immunotherapy may stem from PTEN inactivation, resulting in hyperactivation of the PI3K-AKT pathway. This highlights the necessity of a combination treatment strategy (67). Surgical interventions and conventional chemotherapy often yield unsatisfactory results (68, 69). In recent years, the rapid advancement of immunotherapy techniques, methods, and new drugs has brought new opportunities for the stagnant field of osteosarcoma treatment (70, 71). However, these opportunities come with challenges. Many preclinical studies suggest that immunotherapy may benefit osteosarcoma patients, but clinical trials of single-agent therapies often yield disappointing results, hindering effective treatment. Several promising drugs have faced setbacks, primarily because trials included advanced cases of osteosarcoma that had relapsed or metastasized after conventional chemotherapy. In such cases, patients often have severely compromised immune systems, limiting immunotherapy’s effectiveness. Additionally, the immune microenvironment in tumor patients is complex and dynamic, with tumor cells employing various mechanisms to evade immune therapies, making single-agent approaches often ineffective (36, 72). Meazza et al. confirmed the significance of complete surgical remission and noted a promising (though improvable) survival rate in this patient cohort, highlighting a potential role for immunotherapy using IL-2 and LAK/NK cell activation (73). Boye et al. conducted a phase 2 study of pembrolizumab in advanced osteosarcoma, which was well-tolerated but showed no significant antitumor activity. Future trials should explore combination strategies with immunomodulatory agents in patients selected by molecular response profiles (74). Tang et al. conducted a study showing that amrelizumab combined with adriamycin, cisplatin, methotrexate, and ifosfamide in neoadjuvant treatment for resectable osteosarcoma was safe and tolerable. While this combination may not enhance tumor necrosis rate (TNR), the long-term survival benefits require further investigation (75). Wang et al. found that co-delivering a gel with the αPD-1 checkpoint inhibitor significantly enhances effectiveness in an orthotopic osteosarcoma model. Immunophenotyping data indicate a notable increase in T-cell infiltration and improved anti-tumor immunity at the whole-animal level (76). These findings highlight the necessity for combination therapies that integrate various immunotherapeutic approaches along with surgery, chemotherapy, targeted small molecules, and other novel treatments to create optimal treatment regimens. This strategy is crucial for achieving breakthroughs in the comprehensive treatment of osteosarcoma patients. Numerous clinical trials based on this approach have yielded promising results. Additionally, challenges such as personalized immunotherapy selection, immunotherapy resistance, and drug toxicity should also be addressed (**Figure 8**).

## Strength and limitations

This study presents a comprehensive bibliometric review of the field of immunotherapy for osteosarcoma, encompassing an evaluation of its overall scope, advancements, significant contributions, and emerging trends. Researchers are advised to prioritize recent and highly cited references and topics of interest. However, it is important to acknowledge certain limitations in this bibliometric analysis. Our data source relies solely on Web of Science (WOS), primarily due to the compatibility of bibliometric tools and the feasibility of data processing. We previously considered merging data from multiple databases, such as Scopus, MEDLINE, and Cochrane, to enhance literature coverage. However, we encountered several significant challenges during implementation: differences in citation data formats, the complexity of data deduplication, insufficient tool compatibility, and high resource and time costs. Still, it is worth noting that our analysis was limited to articles exclusively sourced from the WoS Core Collection, potentially limiting the breadth of our findings. Furthermore, the exclusion of recently published articles may be attributed to a temporal delay. Lastly, despite the algorithm’s objective execution of the analysis, we observed an inherent subjective bias in the interpretation of the data.

## Conclusion and future perspectives

The field of immunotherapy for osteosarcoma has undergone significant evolution over time, revealing a notable trend. Notably, China emerged as the leading contributor. Among institutions, Zhejiang University exhibited the highest level of productivity. The author with the highest publication output was Tsukahara, Tomohide from Japan. The article with the most citation was “DOI: 10.1200/JCO.2014.58.0225”. Frontiers in Immunology emerged as the most productive journal. The most frequently occurring keywords in ITFOS research include “osteosarcoma,” “immunotherapy,” and “cancer.” Furthermore, “sequencing,” “prognostic signature,”. and “immune microenvironment” have been identified as emerging research frontiers for the future. To drive notable progress in the comprehensive treatment of osteosarcoma patients, the incorporation of multifaceted therapeutic approaches is imperative. This entails the integration of combination therapies that encompass multiple immunotherapeutic modalities, alongside the inclusion of surgical interventions, chemotherapy, targeted small molecule drugs, and novel therapeutic strategies. The strategic amalgamation of these diverse treatment modalities is poised to have a pivotal impact on advancing the field of osteosarcoma treatment.

## Data Availability

The original contributions presented in the study are included in the article/supplementary material. Further inquiries can be directed to the corresponding author.
